# The Andromonoecious Sex Determination Gene Predates the Separation of *Cucumis* and *Citrullus* Genera

**DOI:** 10.1371/journal.pone.0155444

**Published:** 2016-05-12

**Authors:** Adnane Boualem, Afef Lemhemdi, Marie-Agnes Sari, Sarah Pignoly, Christelle Troadec, Fadi Abou Choucha, Ilknur Solmaz, Nebahat Sari, Catherine Dogimont, Abdelhafid Bendahmane

**Affiliations:** 1 INRA, Institute of Plant Sciences Paris-Saclay (IPS2), CNRS, Université Paris-Sud, Bâtiment 630, 91405, Orsay, France; 2 CNRS, UMR 8601, Laboratoire de Chimie et Biochimie Pharmacologiques et Toxicologiques, Université René Descartes, 75006, Paris, France; 3 Department of Horticulture, Faculty of Agriculture, University of Cukurova, Adana, Turkey; 4 INRA, UR 1052, Unité de Génétique et d’Amélioration des Fruits et Légumes, BP 94, 84143, Montfavet, France; University of Tsukuba, JAPAN

## Abstract

Understanding the evolution of sex determination in plants requires the cloning and the characterization of sex determination genes. Monoecy is characterized by the presence of both male and female flowers on the same plant. Andromonoecy is characterized by plants carrying both male and bisexual flowers. In watermelon, the transition between these two sexual forms is controlled by the identity of the alleles at the *A* locus. We previously showed, in two *Cucumis* species, melon and cucumber, that the transition from monoecy to andromonoecy results from mutations in 1-aminocyclopropane-1-carboxylic acid synthase (ACS) gene, *ACS-7*/*ACS2*. To test whether the *ACS-7/ACS2* function is conserved in cucurbits, we cloned and characterized *ClACS7* in watermelon. We demonstrated co-segregation of *ClACS7*, the homolog of *CmACS-7/CsACS2*, with the *A* locus. Sequence analysis of *ClACS7* in watermelon accessions identified three *ClACS7* isoforms, two in andromonoecious and one in monoecious lines. To determine whether the andromonoecious phenotype is due to a loss of ACS enzymatic activity, we expressed and assayed the activity of the three protein isoforms. Like in melon and cucumber, the isoforms from the andromonoecious lines showed reduced to no enzymatic activity and the isoform from the monoecious line was active. Consistent with this, the mutations leading andromonoecy were clustered in the active site of the enzyme. Based on this, we concluded that active ClACS7 enzyme leads to the development of female flowers in monoecious lines, whereas a reduction of enzymatic activity yields hermaphrodite flowers. *ClACS7*, like *CmACS-7/CsACS2* in melon and cucumber, is highly expressed in carpel primordia of buds determined to develop carpels and not in male flowers. Based on this finding and previous investigations, we concluded that the monoecy gene, *ACS7*, likely predated the separation of the *Cucumis* and *Citrullus* genera.

## Introduction

The majority of angiosperms are hermaphroditic producing exclusively bisexual flowers. Sex determination is a developmental evolutionary process that leads to unisexual flowers in 10% of the species [1]. Monoecious species (5–6%) exhibit male and female flowers on the same plant. Dioecious species (5–6%) have separate male and female individuals. There has been concerted effort in the last few decades to pinpoint the master genes controlling sex determination and to understand the evolutionary forces that drive transitions between sexual morphs [2,3]. In these investigations, several plant families have emerged as model systems [4–7]. The choice of the *Cucurbitaceae* is justified by the wide spread of sex among the species of this plant family. Out of 800 investigated species, 460 are monoecious and 340 are dioecious. Throughout evolution of *Cucurbitaceae*, there have been also numerous shifts between monoecy and other sexual morphs, which makes this plant family a practical model to investigate the molecular mechanisms controlling sex determination.

In monoecious *Cucurbitaceae* species, such as *Cucumis melo*, floral primordia are initially bisexual with sex determination occurring by the selective developmental arrest of either the stamen or the carpel, resulting in unisexual flowers [8]. Cloning and characterization of natural mutations leading to transition from monoecy to different sexual morphs in melon and cucumber has brought new insight to the monoecy sex determination pathway. Three master sex genes, *Monoecious*, *Androecious* and *Gynoecious* genes, interact to explain the gender and the position of the flower on the plant [9–12]. In this model the expression of the carpel inhibitor, *CmWIP1*, encoded by the *gynoecious* gene, is dependent on non-expression of *CmACS11*, encoded by the *androecious gene*. The expression of the stamina inhibitor, *CmACS-7*, encoded by the *monoecious* gene is dependent on non-expression of *CmWIP1*. In monoecious and andromonoecious plants, male flowers develop because of non-expression of *CmACS11* that permits the expression of *CmWIP1*. Female flowers develop on the branches because of expression of *CmACS11* that represses the expression of *CmWIP1*, thus releasing the expression of *CmACS-7* that inhibits the stamina development. If nonfunctional *CmACS-7* is expressed, hermaphrodite instead of female flowers develop. *Androecious* plants are obtained by loss of function of *CmACS11* that leads to expression of *CmWIP1* in all the flowers of the plants. *Gynoecious* plants are obtained by inactivation of *CmWIP1* function. Hermaphrodite plants are obtained by inactivation of *CmWIP1* and *CmACS-7* [9–12].

Although sex determination occurred many times independently during evolution [1,13,14], one would expect that related species use common sexual mechanisms. We previously showed that in melon and cucumber, two species that diverged more than 10 million years ago (Mya) share the same pathway leading to monoecy [10]. For instance the melon and the cucumber *monoecious* gene are orthologs and encode for the rate-limiting enzyme in ethylene biosynthesis, the 1-aminocyclopropane-1-carboxylic acid synthase (*ACS*). In both species, the *ACS* genes, referred to as *CmACS-7* in melon and *CsACS2* in cucumber, are expressed in carpel primordia, and loss of enzymatic activity leads to stamen development [10].

Similarly, the melon and the cucumber *androecious* genes that encode for an ACS are also orthologs [11]. In both species, the *ACS* genes, referred to as *CmACS11* in melon and *CsACS11* in cucumber, are expressed in carpel primordia, and loss of enzymatic activity leads to stamen development [11].

In melon and cucumber, two species of the *Cucumis* genus, our previous studies [9,10] have shown that the sexual transition from monoecy to andromonoecy results from loss-of-function mutations in *CmACS-7* and *CsACS2*, respectively. In watermelon, a monoecious cucurbit, the recessive *a* locus controls the andromonoecious sexual phenotype and acts as *CmACS-7* and *CsACS2* [15]. Recently, a QTL analysis has shown that the major QTL controlling the watermelon andromonoecy is located on chromosome 3 close to the *Cla011230* gene encoding an ACC synthase enzyme [16].

In the present study, we investigated whether components of the monoecy pathway are conserved between the *Cucumis* and *Citrullus* genus that diverged 20 Mya [17]. To test this hypothesis, we cloned the andro*monoecious* (*a*) gene from *Citrullus lanatus* (watermelon) and characterized natural mutations leading to sexual transition between monoecy and andromonoecy. We showed that, like in melon and cucumber, an *ACS* gene, *ClACS7*, inhibits stamina development in female flowers and loss of enzymatic activity leads to stamen development. *ClACS7* is syntenic to *CmACS-7* in melon and *CsACS2* in cucumber. Based on this finding, we concluded that the *ACS7* function is ancestral to the divergence of the *Cucumis* and the *Citrullus* genus.

## Materials and Methods

### Plant material and segregating population

The genetic linkage analysis of the *A* locus and *ClACS7* was carried out using a segregating population obtained from a cross between a monoecious accession, *Citrullus lanatus var*. *citroides*, PI 296341, and an andromonoecious *Citrullus lanatus var*. *lanatus* variety Halep Karasi. The resultant F1 was backcrossed with Halep Karasi.

Plants were grown in the greenhouse in summer at Montfavet, France, under standard agronomic conditions and evaluated for the sex of their flowers at each node along the main stem and side branches.

### Plant genotyping

To identify plants carrying recombination events, plant DNA was extracted from each individual of the backcross segregating population. Determination of the sexual phenotype was performed for all plants. For genotyping, a 556 bp DNA fragment of the *ClACS7* gene was amplified with primers listed in S1 Table and sequenced. Polymorphisms within this fragment were used to determine recombinant alleles.

### Sequence analysis

Multiple sequence alignment of full-length protein sequences of ACS was performed using the ClustalW (http://www.ebi.ac.uk/Tools/clustalw2). Phylogenetic trees were constructed by MEGA6 (http://www.megasoftware.net/index.html) based on the Neighbor-Joining method. The three-dimensional structure predictions were generated using the Geno3D server (http://geno3d-pbil.ibcp.fr) and the visualization was carried out using the Chimera server (http://www.cgl.ucsf.edu/chimera).

### Quantitative RT-PCR experiments

Total RNA was extracted from frozen leaves, stems and flowers with the Trizol reagent (Invitrogen, France). First strand cDNA synthesis and primer design were performed as decribed in [11]. Primers sequences are listed in S1 Table. To check specificity of the designed primers, all amplicons were sequenced and blasted against NCBI database. Quantitative RT-PCR reactions were performed as described in [11]. A negative control without cDNA, technical replicates on three independent synthesis of cDNA (derived from the same RNA sample), and three independent biological experiments were performed in all cases. To compare data from different PCR runs and cDNA samples, CT values for *ClACS7* were normalized to the CT value of *ClActin2* (primers shown in S1 Table). *ClACS7* relative expressions were determined as described in [9].

### *In situ* hybridization

*ClACS7 in situ* hybridisation was performed as described in [18]. Primers used for this experiment are listed in S1 Table.

### Expression and purification of the recombinant ClACS7 isoforms

The BL21(DE3)pLYSS *E*. *coli* strain {*F- ompT hsdSB(rB- m rB) gal dcm (DE3) pLysS (CmR)*} was used for the expression of the enzyme. The plasmid pET15b (Novagen, Germany) encodes T7 promoter, and confers ampicillin resistance. S-Adenosyl Methionine (SAM), Pyridoxal 5’phosphate (PLP) and 5’Adenylic Acid Deaminase from Aspergillus (Deaminase), were purchased from Sigma (France).

The ClACS7 enzymes expression and purification were performed as described in [9], using BL21(DE3)pLYS cells expressing the pClACS7 constructs.

### Enzyme activity assays

Enzyme activity was determined by monitoring the MTA formation by differential spectroscopy recorded on an Uvikon 940 spectrophotometer (Biotek-Kontron Instruments, France) as according to [19]. Namely, specific activity measurement were performed on 3 different enzyme preparations by incubating 60 μM SAM in 100 mM KPhos buffer pH 8.5 (0.2 ml) and deaminase (8 μg for 0.2 ml) in the presence of 5 μM PLP in each quartz cuvette for 3 min at 25°C after addition of the purified enzyme (1 to 2 μg). The conversion of the MTA produced by ACC into an inosine derivative was monitored at 265 nm (Δε = –7740 M^-1^.cm^-1^) and the specific activity was expressed as nmol of MTA formed per min per mg of protein.

## Results

### *Cla011230/ClACS7* is the watermelon orthologue of *CmACS-7/CsACS2*

**Fig 1:**
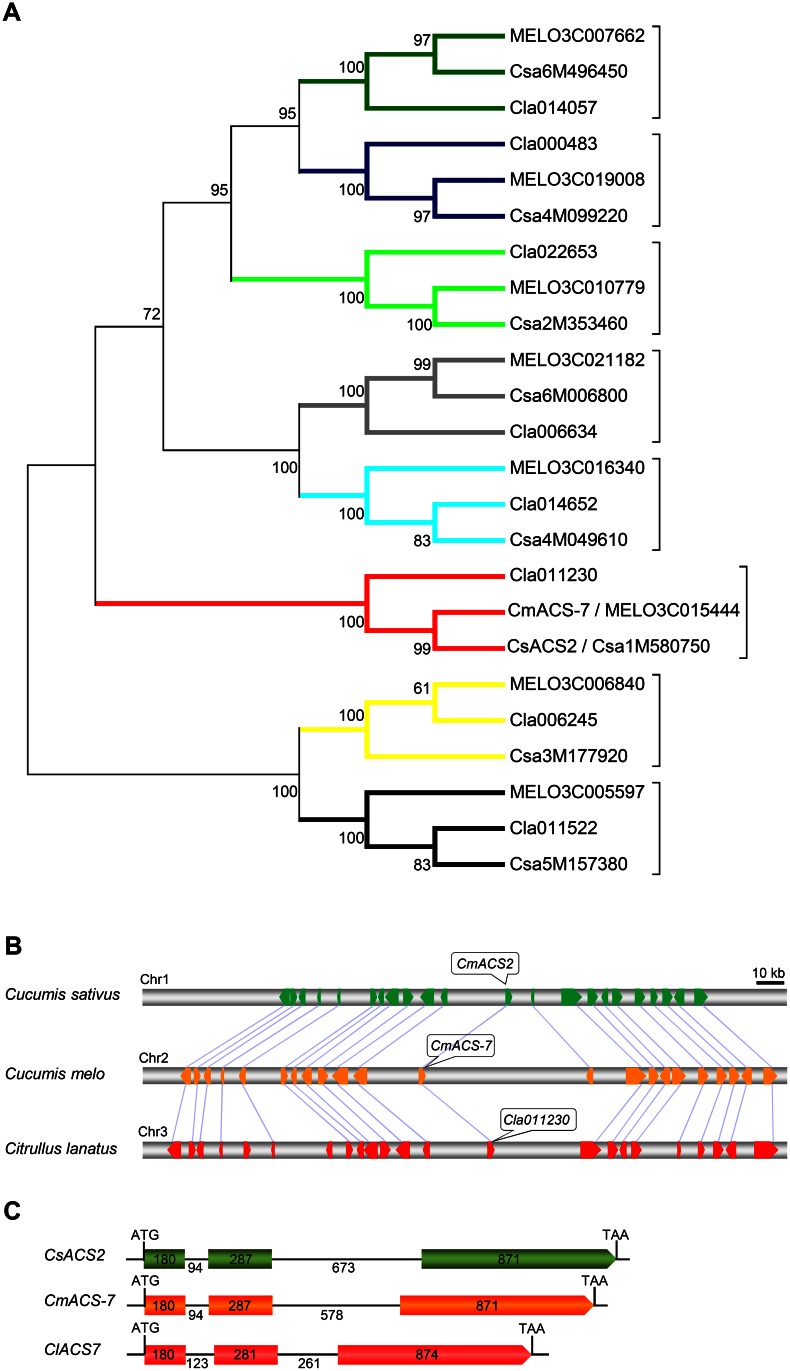
Phylogeny relationship of cucurbit ACS protein family and syntenic and gene structure analysis of watermelon, *ClACS7*, melon, *CmACS-7*, and cucumber, *CsACS2*, genes. (**A**) Using the ClACS7 protein sequence as the query in tBLASTn searches, ACS protein family members were identified from watermelon, cucumber and melon. Protein sequences were aligned and used to generate the neighbor-joining phylogenetic tree with 1,000 bootstrap replicates. The percentage of replicate trees in which the associated taxa clustered together in the bootstrap test are shown next to the branches. ClACS7 clade is indicated in red. (**B**) Syntenic segment between the cucumber chromosome 1 (top), the melon chromosome 2 (middle) and the watermelon chromosome 3 (bottom) harboring *CsACS2* (*Csa1M580750*), *CmACS-7* (*MELO3C015444*) and *ClACS7* (*Cla011230*). Broad horizontal arrows indicated genes and their transcriptional orientations. Syntenic genes are joined by light purple lines. (C) Schematic diagram of *CsACS2*, *CmACS-7* and *ClACS7* gene structure. The numbers, in bps, indicate the size of the exons (filled boxes) and the introns (black lines).

To test whether the genetic determinant controlling monoecy to andromonoecy sexual transition is conserved between the *Citrullus* and *Cucumis* genus and whether the watermelon ortholog of *CmACS-7/CsACS2* could be encoded by the watermelon *A* gene, we isolated all the *ACS* genes from melon, cucumber and watermelon. Both cucurbit genomes contain 8 *ACS* genes and phylogenetic analysis determined that *Cla011230*, hereafter *ClACS7*, *CmACS-7* and *CsACS2* define a monophyletic clade (Fig 1A). *ClACS7* encodes an ACC synthase of 444 amino acids that share 92% amino acid sequence identity with CmACS-7 and CsACS2 and all the polymorphic residues are located in non-conserved positions (S1 Fig). Sequence analysis shows that *ClACS7*, *CmACS-7* and *CsACS2* are highly synthenic and share conserved gene structure with 3 exons and 2 introns (Fig 1B and 1C). Based on this, we concluded that *ClACS7* is most likely the watermelon ortholog of *CmACS-7* and *CsACS2*.

### *ClACS7* co-segregates with the watermelon *andromonoecious* (*a*) locus

**Fig 2:**
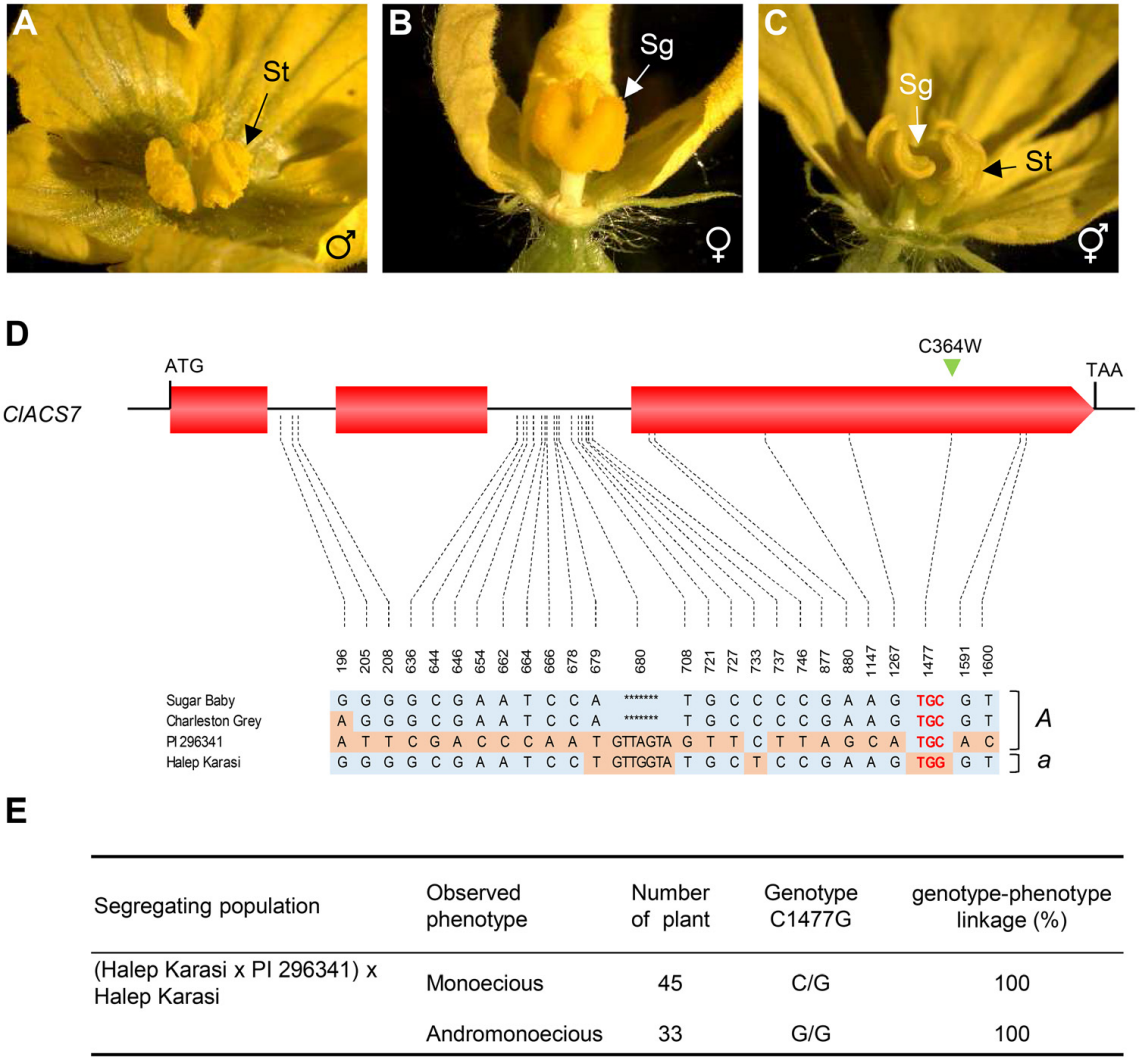
*ClACS7* sequence variation and co-segregation analysis. (**A-C**) watermelon flower types from monoecious and andromonoecious plants. Monoecious watermelon bears male (**A**) and female (**B**) flowers whereas andromonoecious bears male (**A**) and hermaphrodite (**C**) flowers. sg, stigma; st, stamen. (**D**) *ClACS7* sequence variation in monoecious and andromonoecious watermelon lines used in the co-segregation analysis. Polymorphic sites indicated below the gene model were positioned relative to the first nucleotide of the start codon. The red colored nucleotide indicates the C to G transition leading to the amino acid substitution C364W. Genotypes of the parental lines, PI 296341 and Halep Karasi are *AAGyGy* and *aaGyGy*, respectively. *A* and *a* genotypes on the left indicate whether the accessions harbour the dominant *A* or the recessive *a* alleles at the *A* locus. (**E**) Linkage analysis of *andromonoecious* (*A*) locus with *ClACS7*. The observed sexual phenotype, the number of plants, the genotype at the SNP 1477 and the genotype-phenotype linkage frequency are indicated.

To map *ClACS7* relative to the *andromonoecious* (*a)* locus, we first sequenced the *ClACS7* gene in four watermelon lines, Sugar Baby, Charleston Grey, PI 296341 which are monoecious (*A*) harboring male and female flowers(Fig 2A and 2B), and Halep Karasi, an andromonoecious (*a*) line harboring male and hermaphrodite flowers (Fig 2A and 2C). PI 296341 and Halep Karasi were used to generate the mapping population. Among the four accessions, we observed 19 DNA polymorphisms in the introns and 7 in the exons (Fig 2D). Out of the 7 polymorphisms in the coding region, six were silent and only the SNP at nucleotide position 1477 produced a cysteine to tryptophan amino acid substitution at position 364 in the protein (C364W; Fig 2D). The identified C364W substitution was mapped relative to (*a*) sex locus (Fig 2E). In this experiment, a perfect co-segregation of *ClACS7* with the (*a*) locus was obtained in a segregating population of 78 backcross plants (Fig 2E). Based on the functional similarity between monoecy versus andromonoecy in watermelon, melon and cucumber, the high sequence identity between *ClACS7*, *CmACS-7* and *CsACS2* and the co-segregation of the *A* gene with *ClACS7*, we concluded that it is likely that the *A* gene encodes for *ClACS7*.

### Loss of ClACS7 enzymatic activity leads to andromonoecy

**Fig 3:**
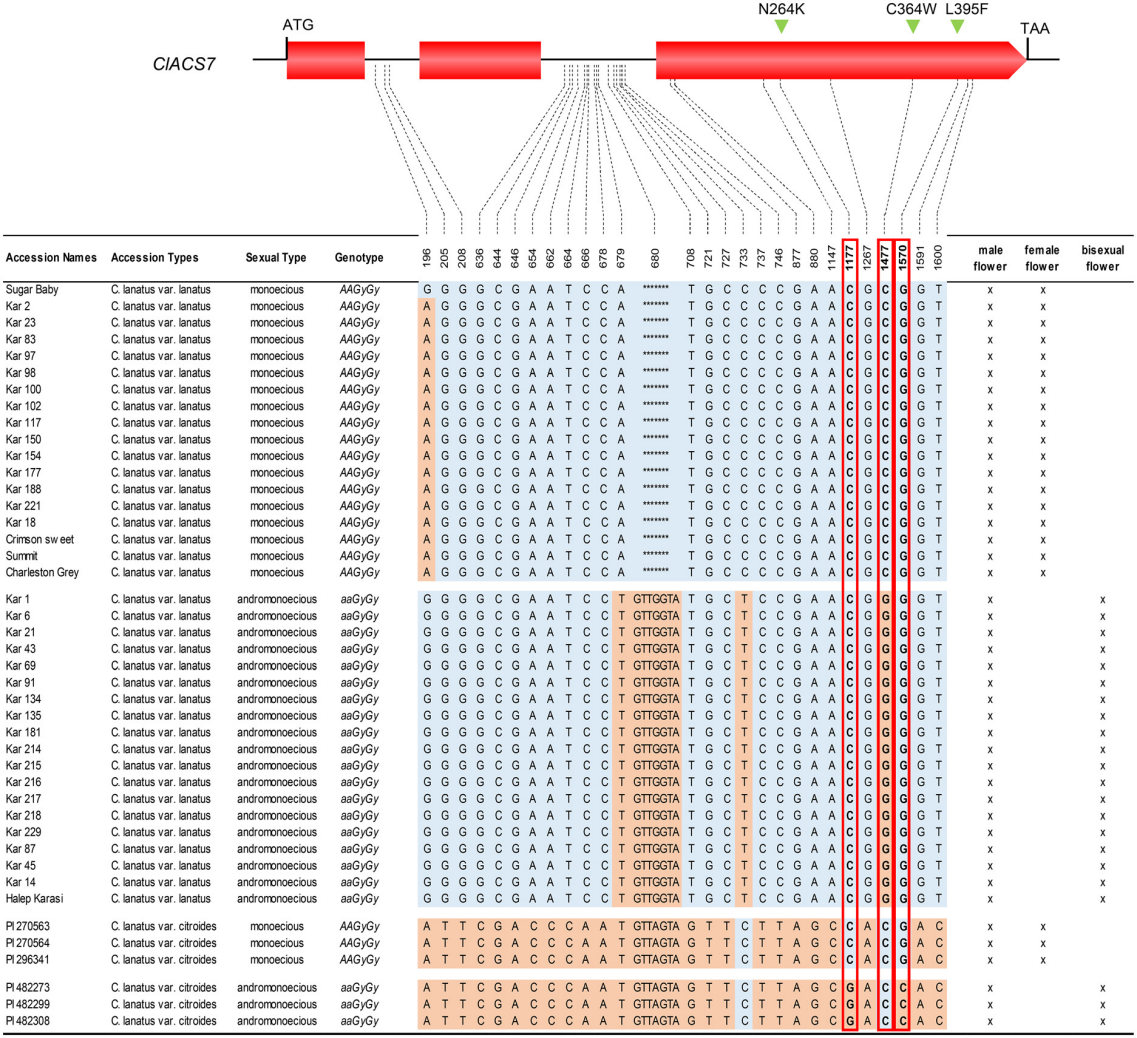
Analysis of *ClACS7* DNA sequence variation in 43 watermelon accessions representing all the sexual morphs of the species. Polymorphic sites indicated below the gene model were positioned relative to the first nucleotide of the start codon. The red boxes point out the nucleotide polymorphisms leading to the missense mutations, N264K, C364W and L395F leading to andromonoecious sexual morph. Presence of male, female or hermaphrodite flowers in the accessions is indicated by (x).

**Fig 4:**
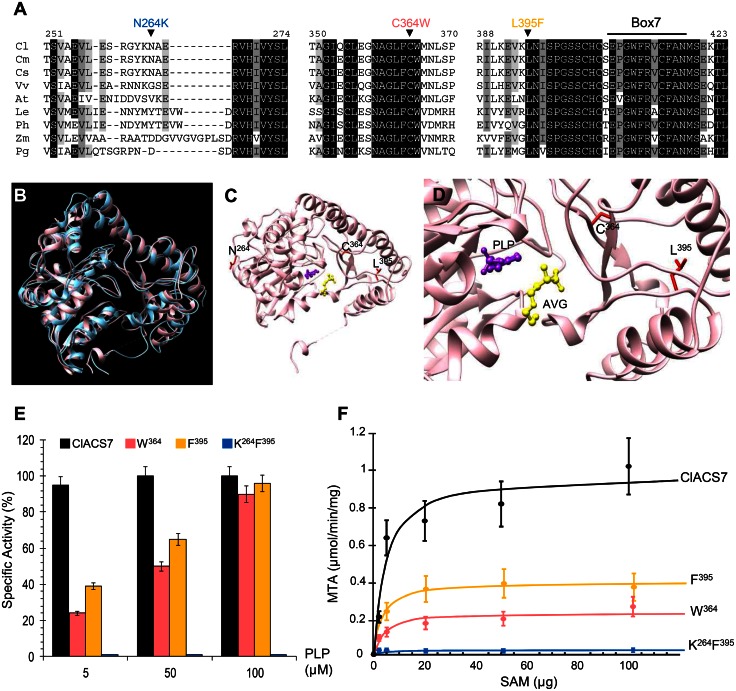
Molecular characterization of ClACS7 alleles. (**A**) Amino acid alignments of ClACS7 isoform with homologous proteins from Cm (*Cucumis melo*), Cs (*Cucumis sativus*), Vv (*Vitis vinifera*), At (*Arabidopsis thaliana*), Le (*Lycopersicum esculentum*), Ph (*Petunia hybrida*), Zm (*Zea mays*) and Pg (*Picea glauca*). Numbers above the alignment indicate the amino acid positions along the ClACS7 protein. Box 7 indicates a conserved domain in ACS proteins. N264K, C364W and L395F amino acid polymorphisms are shown above the alignment. (**B-D**) Model of the three-dimensional structure of ClACS7. (**B**) Superposition of the tomato ACS 3D crystal structure (1IAY.pdb) indicated in blue and the 3D model of ClACS7 indicated in pink. The three-dimensional structure were generated using the Geno3D server (http://geno3d-pbil.ibcp.fr) and the visualization was carried out using the Chimera server (http://www.cgl.ucsf.edu/chimera). (**C** and **D**) Zoom in on the active site of ClACS7. The residues that are polymorphic in the different ClACS7 isoforms are depicted in red. The cofactor PLP is indicated by purple ball and stick. The competitive inhibitor AVG (aminoethoxyvinylglycine) is indicated by yellow ball and stick. (**E** and **F**) Biochemical characterization of ClACS7, W^364^, F^395^ and K^264^F^395^ enzyme isoforms. (**E**) Enzymatic activity of ClACS7 (black bars), W^364^ (pink bars), F^395^ (orange bars) and K^264^F^395^ (blue bars) protein forms. Specific activities were measured on dialyzed enzymes in the presence of 60 μM SAM and various PLP concentrations. (**F**) Initial velocity of the ClACS7 isoforms measured at 50 mM of PLP. Experimental data were fit into a line following Michaelis-Menten equation.

To understand the basis of allelic differences in the *A* gene, we compared the sequence of *ClACS7* in 43 watermelon accessions (Fig 3) selected based on their sexual types. All tested monoecious accessions harbouring the dominant *A* allele presented the same ClACS7 protein sequence. In contrast, analysis of the sequence obtained from the andromonoecious accessions, harbouring the recessive *a* allele, revealed three missense mutations, C364W, N264K and L395F leading to two ClACS7 isoforms, C364W and N264K_L395F (Fig 3). When the closest ClACS7 homologous sequences from different plants were aligned, we observed that the residues C^364^ and L^395^ are conserved across seed plants whereas N^264^ is located in a non-conserved region of the protein (Fig 4A) [20]. Crystallographic studies have determined that C^364^ and L^395^ residues are located in close proximity to the active site (Fig 4B–4D) [21]. The L^395^ residue is located in the conserved box 7 that contains residues involved in the binding of the enzyme substrate SAM (Fig 4B–4D) [21]. Surprisingly, the L^395^ is located at 3 amino acids of the S^399^ and the S399L mutation was previously described as a natural andromonoecious *m* allele in cucumber [10].

To determine whether hermaphrodite flowers in andromonoecious plants are due to loss of a ClACS7 enzymatic activity, we expressed the different forms of the protein as six-histidine tag (His_6_-ClACS7) fusion proteins in *Escherichia coli*. The purified proteins were assessed for enzymatic activity *in vitro* by monitoring 5’-methylthioadenosine (MTA) formation at different PLP concentrations. The K^264^F^395^ isoform was found to be totally inactive (Fig 4E and 4F, Table 1). In the presence of high concentrations of PLP (100μM), the W^364^ and the ClACS7 isoforms share similar biochemical constants (Table 1). However, at a physiological PLP concentration [9], the W^364^ isoform has a reduced enzymatic activity; its *V*_*max*_ is approximately 25% of the *V*_*max*_ of the ClACS7 isoform (Fig 4E, Table 1). For the K^264^F^395^ protein form, to determine whether the amino acid substitution of the conserved residue L^395^ is responsible for the inactivation of the enzyme, we expressed and assessed the enzymatic activity of the F^395^ isoform. Like the W^364^ isoform, at a physiological PLP concentration, the F^395^ isoform has a reduced enzymatic activity whereas at 100μM PLP, the F^395^ and ClACS7 isoforms have similar biochemical constants (Fig 4E, Table 1). The fact that the L395F amino acid substitution does not lead to a complete ClACS7 enzymatic inactivation suggests that the N264K substitution could also reduce enzymatic activity and thus the complete loss of enzymatic activity observed in the K^264^F^395^ isoform is due to a synergic effect of the N264K and L395F substitutions.

**Table 1:** The kinetic parameters, Km and Vm, measured at 5μM and 100μM PLP for ClACS7, W^364^, K^264^F^395^ and F^395^ protein isoforms.

Enzyme	5 μM PLP		100 μM PLP	
	Km (μM)	Vm (nmol.min^-1^.mg^-1^)	Km (μM)	Vm (nmol.min^-1^.mg^-1^)
ClACS7	4.6 ± 1.8	950 ± 80	5 ± 2	1000 ± 50
W^364^	4.5 ± 1.8	240 ± 20	5 ± 3	900 ± 80
K^264^F^395^	nd	≤ 10	nd	≤ 10
F^395^	3.2 ± 0.7	390 ± 10	5 ± 3	960 ± 100

Based on this and because the ClACS7 isoform from the monoecious lines was active and isoforms from the andromonoecious lines showed reduced to no enzymatic activity, we concluded that the loss of ClACS7 enzyme activity is likely the cause of the development of hermaphrodite flowers in watermelon and that ethylene production by ClACS7 is necessary for the developmental inhibition of the male organs in the female flowers of monoecious watermelon. This conclusion is reinforced by recent experiments showing that monoecious watermelons treated with an ethylene biosynthesis inhibitor, the 1-aminoethoxyvinylglycine (AVG), develop hermaphrodite flowers [22]. Moreover, these data show that, in watermelon, the andromonoecy evolved from monoecy, independently in wild *C*. *lanatus* var. *citroides* accessions and in cultivated *C*. *lanatus* var. *lanatus*.

### *ClACS7* is expressed in carpel primordia of female and hermaphrodite flowers

**Fig 5:**
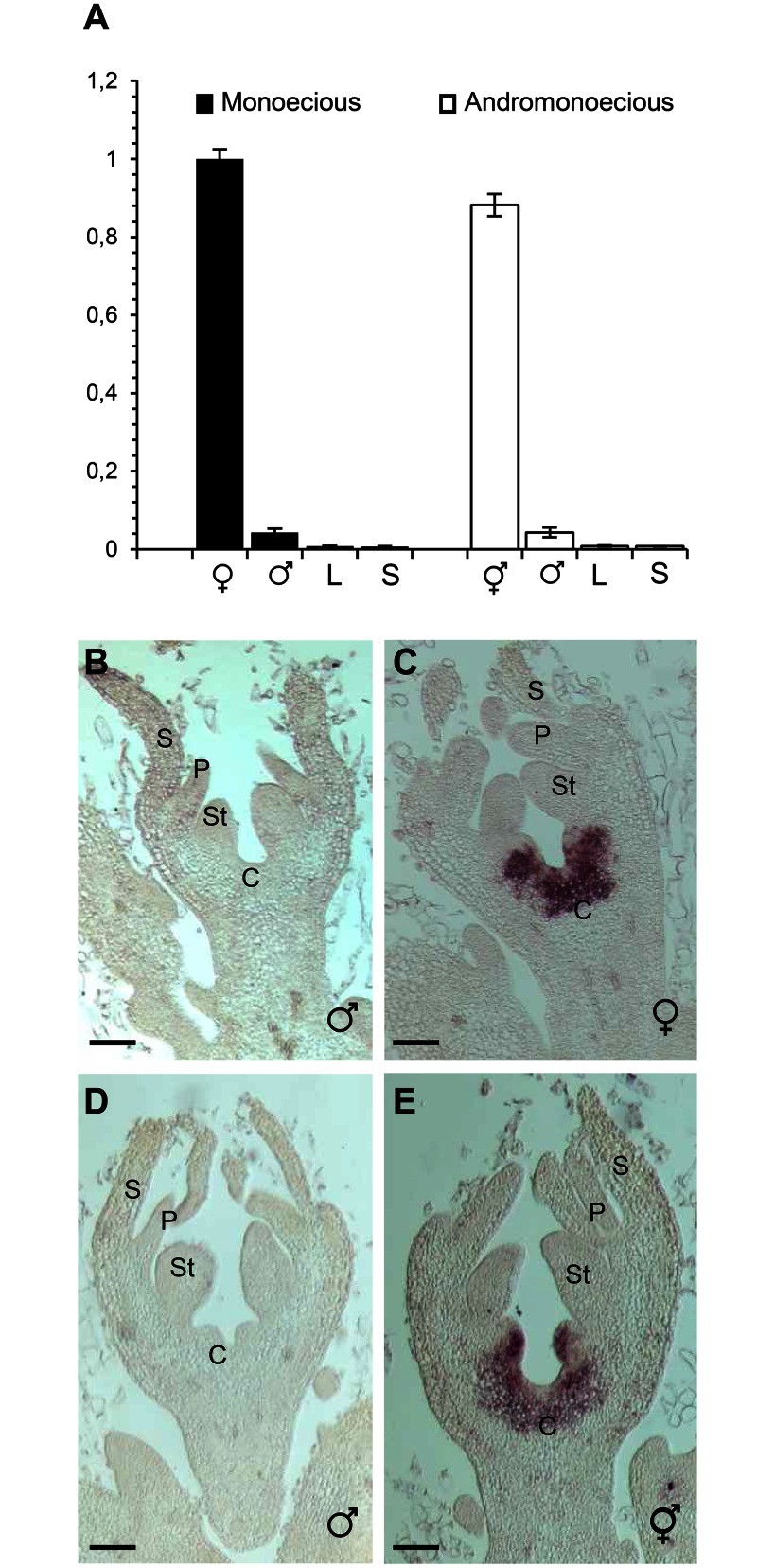
Expression patterns of *ClACS7* in watermelon flowers. **(A)** Quantitative RT-PCR of *ClACS7* in reproductive organs, male or female flower buds from monoecious watermelon and male or hermaphrodite flower buds from andromonoecious watermelon, collected at stage 4, and in the vegetative tissues, leaves (L) and stems (S); shown are the mean ± standard deviation (s.d.) of three biological replicates. **(B-E)** *ClACS7 in situ* expression in flower buds at stages 4 in male (**B**) and female (**C**) flowers of monoecious watermelon and male (**D**) and hermaphrodite (**E**) flowers of andromonoecious watermelon. C, Carpel; St, Stamen; P, Petal; S, Sepal. Bars, 100 μm.

Quantitative real-time PCR analysis demonstrates that *ClACS7* is highly expressed in female and hermaphrodite flower buds and it is weak or undetectable in buds determined to develop into male flowers. No *ClACS7* expression was detected in vegetative tissues (Fig 5A). To investigate in more detail the spatial expression pattern of *ClACS7*, we carried out *in situ* hybridizations. *ClACS7* mRNA was detected in female and hermaphrodite flower buds at stages where buds are not morphologically distinguishable. *ClACS7* mRNA was strongly localised in the carpel primordia (Fig 5C and 5E). In the male flowers of the monoecious or andromonoecious watermelon lines, no *ClACS7* transcript was detected (Fig 5B and 5D).

Since *ClACS7* is expressed in both female and hermaphrodite flowers (Fig 5) and because the loss of ClACS7 activity accounts for the functional variation between the monoecious and andromonoecious watermelon lines (Fig 4), we concluded that ClACS7-mediated ethylene production in the carpel primordia prevents the development of the stamina in female flowers and is also not required for carpel development.

Moreover, since the watermelon *ClACS7*, the melon *CmACS-7* and the cucumber *CsACS2* have the same expression pattern and since the loss of ClACS7, CmACS-7 and CsACS2 enzymatic activities lead to the sexual transition from monoecy to andromonoecy, we concluded that *ClACS7*, *CmACS-7* and *CsACS2* are regulated by mechanisms common to the *Citrullus* and *Cucumis* genus and that ethylene signalling and response mechanisms leading to inhibition of stamina development in female flowers of monoecious plants are conserved in the two genera, *Citrullus* and *Cucumis*.

## Discussion

Most of the species of the Cucurbitales order have unisexual flowers and are mostly monoecious (>75% of the species) bearing both male and female flowers on the same plant. Andromonoecy, a major sexual system in which plants carry both male and perfect bisexual flowers, has evolved independently numerous times [23] and is found in ~ 4,000 species in 33 angiosperm families [24]. Investigations on the genetic inheritance of the different sexual morphs in the *Cucurbitaceae* plant family have described that the andromonoecious trait is controlled by a recessive monogenic locus [5,18,25,26]. In our previous studies, we have demonstrated that the sexual transition from monoecy to andromonoecy, in melon and cucumber, is controlled by the common gene *CmACS-7/CsACS2* [9,10]. In this context, the genetic determinant controlling the monoecy is likely to have been established before the speciation of melon and cucumber, two species of the genus *Cucumis* that have diverged over 10 My ago [17].

In this work, we described the isolation and characterization of the watermelon, *Citrullus lanatus*, *andromonoecious* (*a*) locus and showed that the (*a*) locus encodes for a rate-limiting enzyme in ethylene biosynthesis, *ClACS7*, the orthologous gene of the melon *CmACS-7* and cucumber *CsACS2*. Sequence analysis of monoecious and andromonoecious watermelon accessions revealed two ClACS7 isoforms in the andromonoecious lines, the C364W and the N264K_L395F. Based on the kinetic parameters of the ClACS7 protein isoforms, we concluded that active ClACS7 enzyme leads to the development of female flowers in monoecious lines, whereas a reduction of enzymatic activity yields hermaphrodite flowers in andromonoecious lines. Expression pattern analysis showed that *ClACS7* is specifically expressed in carpel primordia of female and hermaphrodite flowers and no expression was detected in male flowers and the vegetative tissues, leaves and stems. On the basis of enzymatic activity and gene expression studies and as previously described in melon and cucumber [9,10], we concluded that ClACS7-mediated ethylene production in the carpel primordia prevents the development of the stamina in female flowers and is also not required for carpel development.

Altogether, the conserved gene structure, the genomic synteny, the conserved expression patterns and the fact that ClACS7, CmACS-7 and CsACS2 loss-of-function activities lead to hermaphrodite flower development suggest that *ClACS7*, *CmACS-7* and *CsACS2* derive from a common ancestral gene that controls monoecy before the divergence of the *Citrullus* and *Cucumis* genera, 20 My ago [17].

Recently, a study on sex determination in squash, *Cucurbita pepo*, has linked the partial andromonoecy phenotype, i.e. an incomplete conversion of female into hermaphrodite flowers, to the *CpACS27A* gene, an *ACS* gene homologous to *CmACS-7* and *CsACS2* [27]. The similar expression pattern in the carpels of carpel-bearing flowers (female and hermaphrodite) suggest that *ClACS7*, *CmACS-7*, *CsACS2* and *CpACS27A* share common regulatory mechanisms along evolution and that monoecy is controlled by an ancestral gene that precedes the *Cucumis*, *Citrullus* and *Cucurbita* genus divergence 30 My ago [17].

## Supporting Information

S1 Fig Amino acid alignments of ClACS7 with CsACS2 and CmACS-7 homologous proteins from cucumber and melon, respectively. Box 1 to Box 7 indicate conserved domains in ACS proteins. Non-conserved amino acids between ClACS7, CsACS2 and CmACS-7 proteins are highlighted in grey.
(PDF)

S1 Table List of the primers used. (PDF)
