# Supplementary material for: The Andromonoecious Sex Determination Gene Predates the Separation of Cucumis and Citrullus Genera
Source: PLoS One. 2016 May 12;11(5):e0155444. doi: 10.1371/journal.pone.0155444 (PMC4865171; doi:10.1371/journal.pone.0155444)
Supplement: S1 Fig — Box 1 to Box 7 indicate conserved domains in ACS proteins. Non-conserved amino acids between ClACS7, CsACS2 and CmACS-7 proteins are highlighted in grey. (PDF) [file pone.0155444.s001.pdf]

```
                                                                 Box 1
CmACS-7     MAIEIDIEQNPTVELSRIGTSETHGEDSPYFAGWKAYDEDPYNESTNPSGVIQMGLAENQ 60
CsACS2      MAIEIEIEQNSSVELSRIGTSETHGEDSPYFAGWKAYDEDPYNESTNPSGVIQMGLAENQ 60
ClACS7      MAIEIEIEQNPTVELSQIGTSETHGEDSPYFAGWKAYDEDPYNETTNPSGVIQMGLAENQ 60
                                            Box 2
CmACS-7     VSFDLLEEYLEENCEGEGNYLNSGFRENALFQDYHGLFSFRSAMGSFMEEIRGGRAKFDP 120
CsACS2      VSFDLLEEYLEENCEGEGNYLNSGFRENALFQDYHGLFSFRSAMGSFMEEIRGGRAKFDP 120
ClACS7      VSFDLLEEYLEQNCEAEAN--CSGFRENALFQDYHGLLSFRTAMAGFMEEIRGGRAKFDP 118
                               Box 3
CmACS-7     NRVVLTAGATAANELLTFILANPGDALLVPTPYYPGFDRDLRWRTGVKIVPIHCDSSNNF 180
CsACS2      NRVVLTAGATAANELLTFILANPGDALLVPTPYYPGFDRDLRWRTGVKIVPIHCDSSNNF 180
ClACS7      NRVVLTAGATAANELLTFILANPGDALLVPTPYYPGFDRDLRWRTGVKIVPIHCDSSNNF 178
                                         Box 4
CmACS-7     QITPKALEEAYNSAMEMKIKVRGVLITNPSNPLGATIQRSTIEDILDFVTRKNIHLVSDE 240
CsACS2      QITPKALEEAYNSATEMKIKVRGVLITNPSNPLGATIQRSTIEDILDFVTRKNIHLVSDE 240
ClACS7      QITPKALEEAYNTAMAMKIKVRGVLITNPSNPLGATIQRSTIEEILDFVTRKNIHLVSDE 238
                                                     Box 5
CmACS-7     IYSGSVFSSAEFTSVAEVLESRSYKNAERVHIVYSLSKDLGLPGFRIGTIYSYNDKVVTT 300
CsACS2      IYSGSVFSSDEFTSVAEVLESRGYKNAERVHIVYSLSKDLGLPGFRVGTIYSYNDKVVTT 300
ClACS7      IYSGSVFSSAEFTSVAEVLESRGYKNAERVHIVYSLSKDLGLPGFRIGTIYSYNDKVVTT 298
                   Box 6
CmACS-7     ARRMSSFTLISSQTQRFLASMLSNRKFTEKYIKMNRDRLKKRYEMIIEGLRTAGIECLEG 360
CsACS2      ARRMSSFTLISSQTQRFLASMLSNRKFTENYIKMNRDRLKKRYEMIIEGLRTAGIECLEG 360
ClACS7      ARRMSSFTLISSQTQRFLASMLSNRKFTEKYIKMNRDRLRKRYEMIIEGLRTAGIQCLEG 358
                                                               Box 7
CmACS-7     NAGLFCWMNLSPLLKDKK-TKEGEIEIWKRILKEVKLNISPGSSCHCSEPGWFRVCFANM 419
CsACS2      NAGLFCWMNLSPLLKDKK-TKEGEIEIWKRILKEVKLNISPGSSCHCSEPGWFRVCFANM 419
ClACS7      NAGLFCWMNLSPLLKNKKLTREGEIEIWKRILKEVKFNISPGSSCHCSEPGWFRVCFANM 418

CmACS-7     SEKTLHVALDRIRRFMERMKKENEAN 445
CsACS2      SEKTLHVALDRIRRFMERMKKENEAN 445
ClACS7      SEKTLHVALERIHCFMERMRKEDEVN 444
```

Figure S1
