# Supplementary material for: The Andromonoecious Sex Determination Gene Predates the Separation of Cucumis and Citrullus Genera
Source: PLoS One. 2016 May 12;11(5):e0155444. doi: 10.1371/journal.pone.0155444 (PMC4865171; doi:10.1371/journal.pone.0155444)
Supplement: S1 Table — (PDF) [file pone.0155444.s002.pdf]

Analysis	Primer name	Sequence (5' to 3')	Fragment size (bp)
Genotyping	ClACS7_1165_F	CGGCTATAAAAACGCCGAGCGAGTCC	556
	ClACS7_STOP_R	TTAATTAACTTCATCTTCCTTCCTC	
qRT-PCR	ClACS7_F_423	TGCAGCCAATGAGCTTCTTAC	108
	ClACS7_R_876	CCCGTTCTCCATCTCAAATC	
	ClActin2_F	ATGTTGGGGATGAAGCTCAG	187
	ClActin2_R	TTAGCCTTTGGGTTGAGTGG	
in situ experiment	ClACS7_ATG_F	ATGGCGATTGAGATTGAGATTGAGC	1335
	ClACS7_STOP_R_T7	TGTAATACGACTCACTATAGGGCTTA ATTAACTTCATCTTCCTTCCTC	

Table S1 : List of the primers used.
